# Effects of an internet-based cognitive behavioral therapy (iCBT) intervention on improving depressive symptoms and work-related outcomes among nurses in Japan: a protocol for a randomized controlled trial

**DOI:** 10.1186/s12888-019-2221-5

**Published:** 2019-08-07

**Authors:** Kazuto Kuribayashi, Kotaro Imamura, Kazuhiro Watanabe, Yuki Miyamoto, Ayumi Takano, Utako Sawada, Natsu Sasaki, Mariko Suga, Atsushi Sugino, Yui Hidaka, Mako Iida, Mie Sudo, Masahito Tokita, Norito Kawakami

**Affiliations:** 1 Department of Psychiatric Nursing, Graduate School of Medicine, The University of Tokyo, 7-3-1 Hongo, Bunkyo-ku, Tokyo, 113-0033 Japan; 2 Department of Mental Health, Graduate School of Medicine, The University of Tokyo, 7-3-1 Hongo, Bunkyo-ku, Tokyo, 113-0033 Japan; 3 Department of Mental Health and Psychiatric Nursing, Graduate School of Health Care Sciences, Tokyo Medical and Dental University, 1-5-45 Yushima, Bunkyo-ku, Tokyo, 113-8510 Japan; 4 Department of Keio Research Institute at SFC (Shonan Fujisawa Campus), Keio University SFC, 5322 Endo, Fujisawa-shi, Kanagawa, 252-0882 Japan

**Keywords:** Internet-based, Cognitive behavioral therapy, Stress, Depression, Stress management, Prevention

## Abstract

**Background:**

Depression is a major problem among nurses; hence, it is important to develop a primary prevention strategy to manage depression among nurses. This randomized controlled trial (RCT) study aims to investigate the effects of a newly developed internet-based cognitive behavioral therapy (iCBT) program on depressive symptoms, measured at baseline and three- and six-month follow-ups, among nurses in Japan.

**Methods:**

Nurses working at three university hospitals, one public hospital, and twelve private hospitals who meet inclusion criteria will be recruited and randomized either to the intervention group or the control group (planned *N* = 525 for each group). The newly developed iCBT program for nurses consists of six modules, which cover different components of cognitive behavioral therapy (CBT); transactional stress model (in module 1), self-monitoring skills (in module 2), behavioral activation skills (in module 3), cognitive restructuring skills (in modules 4 and 5), relaxation skills (in module 5), and problem-solving skills (in module 6). Participants in the intervention group will be asked to read these modules within 9 weeks. The primary outcome will be depressive symptoms as assessed by the Beck Depression Inventory-II (BDI-II) at baseline, three-, and six-month follow-ups.

**Discussion:**

The greatest strength of this study is that it is the first RCT to test the effectiveness of the iCBT program in improving depressive symptoms among nurses. A major limitation is that all measurements, including major depressive episodes, are self-reported and may be affected by situational factors at work and participants’ perceptions.

**Trial registration:**

This trial was registered at the University Hospital Medical Information Network clinical trials registry (UMIN-CTR; ID = UMIN000033521) (Date of registration: August 1, 2018).

## Background

Most nurses work in stressful settings [1]. Work-related stress among nurses can result from various persistent sources, including heavy workloads, interpersonal conflicts, the emotional impacts of care, lack of reward or control, and shift work [2]. Occupational stress is considered a leading risk factor for a variety of negative health outcomes, including depression [2]. Depression is a major global public health problem due to its high prevalence worldwide and debilitating effects [3]. Nurses can be more vulnerable to depression due to a lack of stress-management skills and/or organizational factors at work [4]. Two surveys conducted in the U.S. reported that the prevalence rate of depression among nurses was 18–35%, higher than among the general population [5, 6]. Depression has deleterious consequences not only on individuals’ biopsychosocial states, but also on organizations’ productivity and functionality [7]. Depression’s biopsychosocial effects impact somatic symptoms [5], quality of life [6], absenteeism [8], work engagement [9], and intention to leave employment [8]. Within organizations, depression affects the quality of care nurses provide [10] and contributes to economic and productivity loss in the workplace [6]. It is essential to maintain nurses’ mental health to ensure their individual well-being, the quality of patient care, and cost-effectiveness. As such, it is important to manage work-related stress and depression among nurses and adverse organizational factors impacting nurses at work as a primary approach to depression prevention.

Concerning stress management for workers in general, a meta-analytic review reported that cognitive behavioral therapy (CBT) interventions significantly reduced depressive or anxiety symptoms [11]. Similarly, two meta-analyses concluded that techniques based on CBT and relaxation were effective in reducing work-related stress among workers and that CBT and relaxation were more effective than other interventions [12, 13]. The estimated effect sizes (Cohen’s d) for CBT and relaxation on work-related stress were 0.68–1.16 and 0.35–0.50, respectively [12, 13]. In the nursing workplace, a Cochrane review reported that compared to no intervention, CBT and relaxation stress-management interventions significantly improved stress-related outcomes, including occupational stress and depressive symptoms, among nurses (standardized mean difference [SMD] = − 0.34 and − 0.59, respectively, at six-month follow-up) [4].

With increased Internet penetration, a new delivery approach for CBT via the Internet has developed, called Internet-based CBT (iCBT). An iCBT program provides users the same principles and techniques as face-to-face CBT programs in a highly structured electronic format consisting of several educational lessons and homework assignments [14]. Benefits of iCBT include its high accessibility and confidentiality. Participants can access the program anytime, anywhere from various Internet-connected devices and review its contents and related feedback as often as they like, which can be useful for users [15]. A literature review stated that the merits of Internet-based interventions in the workplace include the removal of time and location limitations, allowing access to a larger target group at low cost, and increased participant confidentiality, thus reducing barriers to accessing mental health services [16]. The iCBT program could be appropriate for nurses in particular considering that most nurses work in rotating shifts, there are few suppliers of face-to-face CBT programs, like the Employee Assistance Program (EAP), in hospital settings [17], and few nurses receive face-to-face counseling because of barriers to seeking help for mental health [2, 18].

For the working population, stress management based on iCBT has been shown to reduce depressive symptoms. One meta-analysis showed that iCBT interventions had a significant effect on psychological well-being, reducing depressive symptoms (g = 0.25) and increasing work effectiveness, including engagement and productivity (g = 0.26), at post-intervention [19]. Another meta-analysis reported that iCBT programs had a small significant positive effect on workers’ mental health conditions at post-intervention (g = 0.15) [20]. An iCBT intervention might also be effective for nurses in improving depressive symptoms and other work-related outcomes. However, only one randomized controlled trial (RCT) of iCBT has targeted nurses in the U.S. [21]. In that study, the iCBT intervention significantly improved occupational stress among hospital nurses, though its effect on depressive symptoms was not assessed [21]. Although depression has a debilitating effect on nurses, no study has evaluated the effects of an iCBT program on improving depressive symptoms. An RCT must be conducted to verify the effectiveness of an iCBT program in improving depressive symptoms among nurses. Since nurses in the U.S. might be exposed to very different work cultures and stressful situations, experience different thoughts and feelings, and demonstrate different behaviors and coping strategies compared to Japanese nurses, a new iCBT program must be developed for nurses in Japan.

### Objectives and hypotheses

This study has two purposes: (1) to develop a new six-week iCBT program for nurses in Japan and (2) to examine the effectiveness of this iCBT program in improving depressive symptoms as the primary outcome and prevention of the onset of major depressive disorder and improvement in psychosocial work environment, work engagement, and work performance as the secondary outcomes at three- and six-month follow-ups among nurses in Japan.

We hypothesize that the iCBT program will: (1) be associated with lower depressive symptoms at three- and six-month follow-ups among nurses; (2) reduce the risk of major depressive episode (MDE) at the six-month follow-up, and (3) improve the psychosocial work environment, work engagement, and work performance at three- and six-month follow-ups.

## Methods

### Trial design

This study will be a two-arm parallel-group non-blinded RCT with three- and six-month follow-ups. Participants will be requested to complete a baseline online questionnaire. Subsequently, participants meeting the inclusion criteria will be randomly allocated either to the intervention or the control group at a 1:1 ratio. This study’s iCBT program will be provided to the participants in the intervention group for 9 weeks. All surveys will be conducted using online questionnaires. Online follow-up surveys will be implemented at three (immediately after the intervention) and 6 months after the baseline survey. After the six-month follow-up, the iCBT program will be provided to participants in the control group. The study protocol was registered at the University Hospital Medical Information Network (UMIN) Clinical Trials Registry (UMIN-CTR, ID = UMIN000033521). This manuscript was written according to the Standard Protocol Items: Recommendations for Interventional Trials (SPIRIT) guidelines [22].

### Participants

This study will target nurses (i.e., without mental illness) working in a hospital. The participants will be recruited from three university hospitals, one public hospital, and twelve private hospitals (The University of Tokyo Hospital, Yokohama City University Hospital, Yokohama City University Medical Center, Chiba Cerebral and Cardiovascular Center, IMS Katsushika Heart Center, Hokutokai Sawa Hospital, Hoikukai Yokohama Aihara Hospital, Seikeikai Komagino Hospital, Ichiyoukai Youwa Hospital, Hekisuikai Hasegawa Hospital, Inokashira Hospital, Tokyo Musashino Hospital, Sekizenkai Soga Hospital, Sakuunkai Iiduka Hospital, Sakuunkai Yuurin Hospital, and The Salvation Army Booth Memorial Hospital) in Japan. We will recruit nurses from all wards and outpatient sections.

All participants will be required to meet to following inclusion criteria:

1. Registered nurse.
2. Currently employed as a full-time employee by the hospital.
3. Can access the Internet via their own PC, smartphone, or tablet PC.

Participants will be excluded in the following cases:

1. Nurses who are going to take a leave of absence for any reason or leave the hospital during the six-month study period.
2. Practical nurse or nursing aide.
3. Non-regular, part-time, or temporary nurses.
4. Diagnosed with major depressive disorder during the previous month (assessed via the online version of the WHO-Composite International Diagnostic Interview (WHO-CIDI v 3.0) [23].
5. Diagnosed with lifetime bipolar disorder (WHO-CIDI v 3.0).
6. Sick leave for 15 or more days in total due to own health problems during the past 3 months.
7. Those who currently receive treatment, including psychotherapy, from a mental-health professional.
8. Strong suicidal ideation (score of 3 on item no. 9 of the Beck Depression Inventory-II (BDI-II)).

### Procedure

**Fig. 1:**
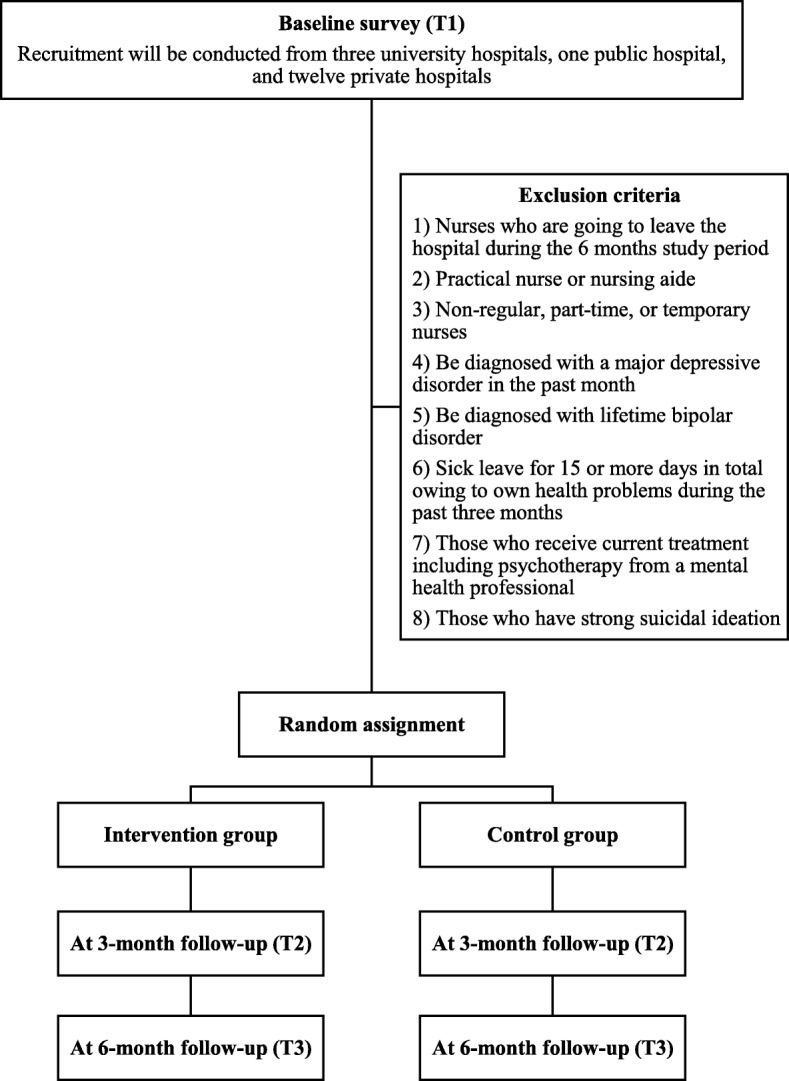
Participant flowchart

Figure 1 shows the participant flowchart for this trial. The researchers will email an invitation via the nursing departments to the nurses who work at three university hospitals, one public hospital, and twelve private hospitals. The email will include an outline of the study and the URL of a website that will fully describe the study. Candidates will click the URL and read a full explanation of the purpose and procedures of the study on the website. Nurses who are interested in participating will be asked to mark the consent option and type their name, the name of the hospital where they are working, and their email address to give their consent. This consent information will be sent to the research center and preserved. Nurses who are not interested in participating will be asked to close the website. Subsequently, participants who submitted the consent information will receive an email containing the URL of the baseline survey. They will click the URL and input their baseline information. After completing the baseline questionnaire, the participants who meet eligibility criteria will be randomized either to the intervention or to the control group. The researchers will inform the participants of their group assignment. In order to avoid delay between the randomization and the beginning of intervention, the researchers will assign the participants to a group as soon as the baseline survey is completed. The researchers will send an email containing the iCBT program’s URL and an ID and password to the participants in the intervention group. Participants in the intervention group will click the URL, enter their own ID and password, and receive the iCBT program. They will be asked not to share their ID, password, and the iCBT program contents with others. Those in the control group will receive the iCBT program after the six-month follow-up.

### Intervention program

This study’s iCBT program for nurses was developed by extensively modifying a previous iCBT program aimed at improving depressive symptoms among newly graduated nurses [24]. The iCBT program for newly graduated nurses was developed through extensive modification the iCBT program for workers developed by Imamura et al. (2014) [25]. To develop the iCBT program for newly graduated nurses, we interviewed experienced nurses working in various wards to identify the most common stressful situations they experienced as newly graduated nurses as well as their thoughts, feelings, behaviors, and coping strategies in such situations. These interviews yielded a number of common stressful situations—lack of confidence in new knowledge and skills, being scolded by senior nurses, difficulty in communicating with and reporting to a senior nurse, heavy workloads, and shift work—and associated distorted cognitive patterns. Similarly, in developing this study’s iCBT program, the content had to reflect the normal work situations and culture experienced by nurses in Japan. Thus, hearings were conducted with nurses in Japan who were working in various wards and outpatient departments in hospitals as full-time employees to identify common stressful situations and nurses’ thoughts, feelings, behaviors, physical feelings, and coping strategies in such situations. Based on these hearings, we modified case stories to reflect common and major stressors (i.e., when changing wards or entering a workplace, nurses face stressful situations, such as an unfamiliar environment, new knowledge and skill requirements, and interpersonal problems) and replaced one module (on communication assertiveness) with another (behavioral activation) because nurses acquire communication skills over time.

Participants allocated to the intervention group will study the iCBT program. Called “*Useful mental health solutions for work and everyday life,*” the program is a six-module internet-based training program that provides stress-management skills based on CBT via a manga story. The use of manga in learning has been reported to 1) stimulate interest in learning, 2) facilitate understanding of the contents, and 3) maintain the motivation to learn [26, 27]. Some existing iCBT programs (e.g., THIS WAY UP [28]; SPARX [29]) utilize comics and manga characters to enhance understanding and users’ motivation to learn the contents. This study’s iCBT program will also use comics and manga characters to increase users’ understanding and motivation. Each week, a new module will be delivered. At the end of each module, the participants will be asked to submit homework that will ask them to apply their learned skills to nursing or daily life situations that they personally perceive as stressful. This homework will be voluntary. Nurses will be able to complete and submit homework only on a PC or a tablet PC, as we lacked sufficient resources and funding to make this function available on smartphones. Each module will require about 30 min to complete, including homework. Participants can receive the program at any time and place via the Internet. Nurses in the intervention group will be asked to read the six modules within 9 weeks after the program starts. After 9 weeks, researchers will close all modules.

Table 1 presents the contents of this study’s iCBT program. The iCBT program will consist of six modules, each covering a different component of CBT: transactional stress model (in module 1), self-monitoring skills (in module 2), behavioral activation skills (in module 3), cognitive restructuring skills (in module 4 and 5), relaxation skills (in module 5), and problem-solving skills (in module 6).

**Table 1:** Contents of the iCBT program for nurses

Module number	Title	Contents
1	Mechanism of stress	Learning about a transactional stress model and noticing stress responses and stressors
2	Tips on self-case formulation using a CB model	Learning about a CB model and the tips for self-case formulation using this model
3	Tips to increase your activity	Learning about behavioral activation skills
4	Cognitive restructuring, part 1	Learning about Beck’s cognitive model and the tips on self-monitoring based on this model
5	Cognitive restructuring, part 2 and relaxation	Learning about cognitive restructuring skills and relaxation skills
6	Effective problem-solving technique	Learning about problem-solving methods

This study’s iCBT program will contain two supplemental modules. One is the introduction module. When participants in the intervention group begin the program for the first time, they will be urged to complete the introduction module, which provides a general description of the program. The introduction module will consist of a brief description of stress and an explanation of the operability of this iCBT program. Participants will be informed about the negative effects of stress on physical and mental health. Furthermore, they will be shown how to use the internet-based program. Another module will contain a mood self-assessment using Kessler’s Psychological Distress Scale (K6) [30, 31], which we call the “mood checker.” Participants can assess their own psychological health at any time using this module. The detailed explanation of the operability and self-monitoring are likely to improve the attrition rate, a limitation of applying internet-based interventions [32, 33]. According to previous systematic reviews, the mean attrition rate in internet-based psychological intervention programs conducted in the workplace is 23%, although it varies significantly across studies [19]. Low literacy in the use of internet-based programs and limited feedback regarding participants’ status could lead to dropout [32–34]. Improving attrition rates in internet-based intervention programs increases the frequency with which users engage with the contents [19], which may be an important determinant of effectiveness [35].

Additionally, this iCBT program includes two supports. One is feedback support from researchers proficient in CBT on homework. Participants who submit their homework will receive feedback to encourage their understanding. Five researchers in the department of mental health and psychiatric nursing will provide feedback. To maintain high feedback quality, the researcher (KK) will offer simple instructions and training along with a manual that will include an example of feedback comments. Another is question support. Participants will be able to ask the researcher (KK) questions about the program or its contents. The researcher will answer the questions in a timely manner. Supported iCBT programs have been reported to be more effective in the management of depressive symptoms compared to self-guided iCBT programs [15, 20]. Additionally, supported e-health programs have been reported to have lower attrition rates than those without support [36]. The two-way supports offered by this program may enhance adherence to and the effectiveness of this program.

#### Module 1: mechanism of stress

In the first module, participants will learn about a transactional stress model [37]. According to the model, an individual’s reaction to stressors, in part, depends on their own appraisal of the stressor. Learning the transactional stress model becomes the basis for understanding what they are experiencing in a stressful situation. Ms. Rino, a clinical psychologist, will teach the relationship between a stressor and the stress reaction to the nurse “Ms. Abe,” another character. Homework for this module asks the participants to list their own stressors and stress reactions.

#### Module 2: tips on self-case formulation using a CB model

In this module, participants will learn about a cognitive behavioral (CB) model and receive tips on self-case formulation. Case formulation is a method to understand the client’s problem individually [38]. Case formulation using a CB model has two main advantages. First, it helps us determine what the client experiences in stressful situations. Case formulation using this model considers the client’s problem from five perspectives (situation, thoughts, emotions, physical feelings, and behavior) [39] to facilitate understanding of the client’s problem. Another advantage is that it helps us consider effective solutions to vicious patterns that afflict a client. Ms. Rino will explain the method of case formulation using an example of a nurse with occupational stress. Additionally, she will offer tips on self-case formulation. This module’s homework includes self-case formulation concerning a participant’s stressful event.

#### Module 3: tips to increase your activity

In this module, participants will learn about a behavioral activation (BA) technique. BA is a process to increase pleasurable and rewarding activities [40]. A meta-analysis reported that BA improved depressive symptoms among clinical populations (SMD = 0.74, 95% confidence interval [CI] = 0.56–0.91) [41]. Another meta-analysis reported that BA, often combined with CBT interventions, prevents depression in non-clinical populations [42]. In the CB model, thoughts, emotions, physical feelings, and behavior interact. Increasing pleasurable and rewarding activities can reduce negative thoughts and moods and improve physical responses to stress. Because pleasurable and rewarding activities differ across individuals, BA includes a process of developing and individually suited activity schedule. Ms. Rino will explain the theory of BA, and she will demonstrate how to plan pleasurable and rewarding activities. This module’s homework asks the participants to develop and schedule an activity.

#### Module 4: cognitive restructuring part 1

In this module, participants will first learn about the “cognitive ABC (Activating/Actual event, Belief, and Consequence) model” [43]. The model explains the perception of an event as stressful rather than the event itself induces stress reactions. Ms. Rino will use a common stressful situation in the nursing field as an example to explain the model. By learning about the model, participants will notice that if their thoughts change, their emotions can improve. Subsequently, they will learn about Beck’s cognitive model and receive tips on self-monitoring based on this model. The model premises that dysfunctional schemas shape an individual’s automatic thoughts, thus influencing the individuals’ emotions [44]. Participants will recognize the importance of identifying their own automatic thoughts that cause a negative mood. This module’s homework includes an exercise to monitor participants’ own automatic thoughts and emotions in a stressful situation.

#### Module 5: cognitive restructuring part 2 and relaxation

In this module, participants will learn about a cognitive restructuring skill useful in cognitive therapy. This cognitive restructuring skill is a major standard component of CBT [45]. A meta-analysis showed that cognitive therapy significantly reduced depressive symptoms among patients with major depressive disorder compared with no intervention [46]. In addition, a previous Cochrane review reported that CBT resulted in significant improvements in stress-related outcomes, including depressive symptoms, compared to no intervention among nurses (SMD = 0.34) [4]. Ms. Rino will teach participants how to change an automatic maladaptive thought into a rational thought. Participants will also learn about a relaxation technique. A Cochrane review reported that relaxation significantly improved stress-related outcomes, including depressive symptoms, compared to no intervention among nurses (SMD = 0.59) [4]. In this module’s homework, participants will apply a cognitive restructuring skill to address their stressful situations.

#### Module 6: effective problem-solving skill

In this module, participants will learn a problem-solving skill. A meta-analysis reported that problem-solving was effective in the treatment of depression in adults, with Cohen’s d = 0.83 (95% CI: 0.45 to 1.21) compared with control conditions [47]. Additionally, a previous Cochrane review reported that a problem-solving skill, often included in CBT interventions, was effective in the primary prevention of stress-related outcomes, including occupational stress and depressive symptoms, among healthcare workers [4]. Theoretically, a rational problem-solving skill must (1) clarify a problem to be addressed, (2) generate potential solutions to cope with the problem, (3) evaluate each solution, (4) implement selected solutions, and assess the result [48]. Ms. Rino will teach the nurse, Ms. Abe, each step in the process. This module’s homework includes a problem-solving exercise involving stressful situations that participants encounter in their workplace or everyday life.

### Intervention group

Participants in the intervention group will be expected to complete one module a week. Each week, one module will be delivered, and all modules will be delivered in 6 weeks after the program starts. Every Monday, the research center will send a reminder email to those who have not completed each module.

### Control group

Participants in the control group will receive the iCBT program after the six-month follow-up. The participants in the intervention group and the control group will be able to undergo treatment as usual (TAU), such as stress management education or training provided by the nursing association. Few lectures address the knowledge and skills involved in CBT in such education or training. After the six-month follow-up, the iCBT program will be provided to participants in the control group for 9 weeks upon their request.

### Outcomes

Table 2 shows an assessment schedule of this study’s outcome measures. All primary and secondary outcomes except for the onset of MDE will be assessed at the baseline (T1), three-month follow-up (T2; immediately after the completion of the intervention), and six-month follow-up (T3). The onset of MDE will be assessed at the baseline and six-month follow-up. Only participants in the intervention group will be assessed regarding the usability of the iCBT program and their satisfaction with it at the three-month follow-up. Information contamination will be assessed at the three- and six-month follow-ups. All data will be collected using web-based self-report questionnaires. At the three- and six-month follow-ups, the research center will send at least two reminder emails to those who have not responded to the questionnaires.

**Table 2:** Assessment schedule of the outcome measures for this study

Measurement	Aim	Baseline (T1)	3-M F/U (T2)	6-M F/U (T3)
Primary outcome				
BDI-II	Severity of depression	x	x	X
Secondary outcomes				
CIDI	The onset of MDE	x		x
JCQ	Psychosocial work environment	x	x	x
UWES	Work engagement	x	x	x
HPQ	Work performance	x	x	x
Sick leave days	Sick leave days during the past 3 months	x	x	x
Intention to leave	Intention to leave their organization	x	x	x
Knowledge and self-efficacy	Current knowledge of and self-efficacy in relation to the five components of the CBT program	x	x	x
Process evaluation				
Usability and satisfaction	Usability and satisfaction of the iCBT program		x	
Others				
Contamination	Contamination of information		x	x

Note: *BDI-II* Beck Depression Inventory II, *CIDI* WHO-Composite International Diagnostic Interview, *MDE* Major Depressive Episode, *JCQ* Job Content Questionnaire, *UWES* Utrecht Work Engagement Scale, *HPQ* WHO Health and Work Performance Questionnaire, *CBT* Cognitive Behavioral Therapy

### Primary outcome

#### Depressive symptoms

The Beck Depression Inventory-II (BDI-II) is a self-report inventory that evaluates depression severity. BDI-II includes 21 items measuring depressive symptoms, such as sadness, pessimism, suicidal thoughts or wishes, tiredness or fatigue, loss of energy, and loss of pleasure [49, 50]. Each item is scored on a four-point scale ranging from 0 to 3. Higher scores indicate higher depression severity. The Japanese version of the BDI-II developed by Kojima et al. (2002) has shown the same internal reliability and validity as the original version [51].

### Secondary outcomes

#### Incidence of MDE

The onset of MDE will be assessed using the online version of the Japanese WHO-CIDI v 3.0 depression section based on the DSM, Fourth Edition, Text Revision (DSM-IV-TR) criteria [23, 52]. CIDI is a structured diagnostic interview, administered by trained individuals to the general population [53]. The Japanese version of the WHO-CIDI v 3.0 was developed by Kawakami et al. (2005), and its validity in diagnosing MDE has been confirmed [23]. The online version asks respondents the same set of questions using the skip logic of the depression section. A computer program with an algorithm specific to WHO-CIDI v 3.0 automatically diagnoses MDE. The online version has been shown to be consistent in the clinical diagnosis of MDE, [54] and the reliability of this version has been confirmed in a 1-year test-retest survey [55]. While WHO-CIDI v 3.0 was originally designed to generate a diagnosis based on the DSM-IV-TR criteria, its instrument can also generate a diagnosis of MDE according to DSM-5 criteria [56].

#### Psychosocial work environment

Psychosocial work environment will be evaluated using the Japanese version of the Job Content Questionnaire (JCQ) [57, 58]. The JCQ is a 22-item self-administered instrument designed to measure the psychosocial environment in the workplace, based on Karasek’s Job Demands-Control model [59] and the Demand-Control-Support model [60]. The JCQ scales consist of psychological demands (5 items), job control (9 items), supervisor support (4 items), and coworker support (4 items). Each item is scored on a scale from 1 (*strongly disagree*) to 4 (*strongly agree*). The reliability and validity of the Japanese version of the JCQ are acceptable [57].

#### Work engagement

Work engagement is a new concept from a positive perspective of mental health. Work engagement will be measured using the short form of the Japanese version of the Utrecht Work Engagement Scale (UWES-J) [61, 62]. UWES-J consists of nine items measuring three subscales (i.e., vigor, dedication, absorption). Each item is assessed on a seven-point scale from 0 (*never*) to 6 (*always*). The total score is calculated from all nine items. The reliability and validity of the UWES-J have been established [62].

#### Work performance

Work performance will be evaluated using one item of the WHO Health and Work Performance Questionnaire (HPQ) [63]. The HPQ is a self-report measure designed to estimate the workplace costs of health problems. In this study, participants will be asked to rate their overall work performance during the past 4 weeks. Items are scored on an 11-point scale ranging from 0 (*worst possible performance*) to 10 (*best possible performance*). A high score indicates a high degree of work performance.

#### Sick leave days during the past three months

Participants will be asked to report their number of sick leave days during the past 3 months.

#### Intention to leave

Intention to leave will be measured using the Japanese version of the four-item scale used by Geurts, Schaufeli, and De Jonge [64, 65]. The scale is a self-report inventory assessing the extent to which respondents expressed an intention to leave their organization during the last month. Each item is scored on a scale from 1 (*strongly disagree*) to 5 (*strongly agree*). Higher scores indicate greater intention to leave. Reliability and validity of the Japanese version have not yet been determined.

#### Knowledge and self-efficacy

Participants will be asked to rate their current knowledge of and self-efficacy in the five CBT components of the program (stress management, behavioral activation, cognitive reconstruction, relaxation training, and problem solving). The items assessing knowledge will start with the question, “How much knowledge do you have about…” while the items assessing self-efficacy will start with “How confident are you that you can do…” Each item will be scored on a 5-point scale ranging from 0 (*none*) to 4 (*enough*). Higher scores indicate greater knowledge of and self-efficacy in CBT components.

### Psychological distress

Psychological distress will be measured only at baseline using the Japanese version of the K6 [30, 31]. Participants will be stratified at baseline based on K6 score (5 or greater or less than 5). The K6 is a self-report inventory assessing the frequency with which respondents have experienced symptoms of psychological distress during the past 30 days. Each item is scored on a scale from 0 (never) to 4 (all of the time). Higher scores indicate more severe psychological distress [66]. The internal reliability and validity of the Japanese version of the K6 are acceptable [30].

### Process evaluation

#### Usability of and satisfaction with the iCBT program

The usability of and satisfaction with the iCBT program will be assessed using original questions. Sample questions include, “How easy was it to understand the program?” and “How satisfied were you with the program as a whole?” Each item will be scored on a 5-point scale ranging from 0 (*none*) to 4 (*enough*). In addition, participants will be asked which device they used most frequently.

#### The number and types of modules and homework

The number and types of modules and homework will be evaluated using the e-learning system records.

### Contamination of information

Contamination of information will be assessed at three- and six-month follow-ups through one original question answered on a dichotomous yes/no scale: “During the past three months, have you received information on stress management from your colleagues who used this study’s iCBT program?”

### Demographic characteristics

Demographic data, including age, gender, marital status, education, employment status, position, years of experience as a nurse, and overtime hours during the past month will be collected.

### Sample size calculation

This study’s iCBT program will include mental health professionals’ support. Previous meta-analyses of iCBT aimed at improving depressive symptoms among workers, both with or without supports, yielded effect sizes of 0.16–0.25 [19, 20]. Programs with supports have been reported to be more effective compared to self-guided iCBT programs [15, 20]. To detect a small effect size of 0.2 or greater at an alpha error probability of 0.05 and a power (1-β) of 0.80 using G*Power V.3.1.9.2 [67], a sample size of 394 participants per arm would be required. Anticipating a dropout rate of 25%, the necessary sample size would be 525 participants per arm.

### Randomization

After the baseline survey, eligible participants will be randomly assigned either to the intervention or control group using stratified permuted-block randomization, with a random block size of two. Participants will be stratified based on K6 score (5 or greater or less than 5) at baseline survey and worksite. The intervention effect may differ according to the severity of the psychological distress at baseline. The K6 cut-off score will be based on the optimal cut-off value (i.e., 4/5), which was used to separate patients with mood disorders in a previous study [66]. An independent biostatistics researcher will generate a stratified permuted-block random table. This table will be password-protected and concealed to other researchers. The researcher (KK) will carry out enrollment and another independent researcher will conduct allocation. During the random assignment, only the researcher will be able to access the stratified permuted-block random table.

### Statistical methods

#### Clinical efficacy

For primary and secondary outcomes, except the onset of MDE, a mixed model for repeated measures conditional growth model analysis will be conducted on an intention-to-treat basis. We will analyze a group (intervention and control) * time (baseline and three- and six-month follow-ups) interaction as an indicator of intervention effect. Missing values will be imputed by applying maximum likelihood estimation using the MIXED procedure. As the surveys will be conducted via the Internet, missing values will not occur unless the participants do not answer the questions. Cohen’s d between groups and 95% confidence intervals will be calculated at each assessment term to assess effect size. The values of 0.2, 0.5, and 0.8 are generally interpreted as small, medium, and large effects, respectively [68]. In addition, we will also calculate the Number Needed to Treat (NNT) because of the difficulty of interpreting the effect size from a clinical perspective. The NNT is the number of persons who must be treated to yield a difference in the onset of an event in one person compared to the control group. In this study, the NNT to alleviate depressive symptoms to achieve improvement in one individual from subthreshold depression will be calculated. Based on the cut-off BDI-II score, this improvement was defined as a change from a score of 14 or more to a score of 13 or less.

Regarding the onset of MDE, a survival analysis will be performed to compare the intervention and the control groups in terms of their mean survival time without MDE to evaluate the effectiveness of the intervention. The survival time will be calculated for each participant as months from the baseline survey to the onset of MDE or the end of six-month follow-up survey. The cumulative incidence ratio of MDE and event-free survival ratio at the six-month follow-up will be estimated using the Kaplan-Meier method. To test the difference in survival probability between the intervention and the control group, a log-rank test will be performed.

#### Subgroup analysis

Since the effect of the iCBT program may differ according to specific population, the effect will also be analyzed by subgroups. Participants will be divided according to their initial severity of psychological distress. We will treat high/low psychological distress (i.e., participants who scored 5 or more on K6 at baseline) as one stratification factor and perform subgroup analysis. In addition, newly graduated nurses (i.e., less than 1 year of nursing experience) have been reported to have higher depressive symptoms compared to veteran nurses [7, 69, 70]. We will treat participants with more/less than 1 year of nursing experience as another stratification factor and conduct subgroup analysis.

### Data monitoring

A data monitoring committee (DMC) will consist of at least one independent member. The trial will be checked by the DMC. After randomizing the participants, the DMC will meet every 3 months to review a report created by the researchers to monitor the progress of recruitment and data collection (e.g., completion rates for each follow-up).

### Data confidentiality

After the surveys, the collected data will be transferred to a password-locked stand-alone PC. The PC will be kept in a locked research room at the Department of Psychiatric Nursing in the Graduate School of Medicine at the University of Tokyo. The collected data will be saved as linkable anonymous data with sequentially allocated numbers. The researchers will be able to access the data.

### Dissemination of research findings

The findings of this study will be published in peer-reviewed international journals. We will also present the results and findings at related research conferences, academic symposiums, and seminars.

## Discussion

The greatest strength of this study is that it is the first RCT, to the best of our knowledge, to evaluate the effect of an iCBT program on improving depressive symptoms among nurses. If successful, the iCBT program for nurses could be a promising tool to improve depressive symptoms as a primary prevention. Additionally, this study also aims to add to the evidence on the effect of iCBT programs on positive health outcomes (i.e., work engagement and work performance) among nurses.

Nevertheless, this study has some limitations. All data, including MDE measurement, will be measured by self-report, which may be affected by situational factors at work and participants’ perceptions. In addition, due to limited resources and funding, the participants will not be able to submit their homework via smartphone, which may limit the effect of our intervention program. Moreover, there is the possibility that participants in the control group will obtain information about the iCBT program from participants in the intervention group in the same ward or workplace. This contamination may weaken the intervention effect.

## Data Availability

Those interested in this study will communicate all outcomes and findings through publications.

## Abbreviations

BA: behavioral activation
BDI-II: Beck Depression Inventory-II
CB: cognitive behavioral
CBT: cognitive behavioral therapy
CI: confidence interval
DMC: data monitoring committee
DSM-IV-TR: DSM, Fourth Edition, Text Revision
EAP: Employee Assistance Program
HPQ: WHO Health and Work Performance Questionnaire
iCBT: Internet-based cognitive behavioral therapy
JCQ: Job Content Questionnaire
MDE: major depressive episode
NNT: Number Needed to Treat
RCT: randomized controlled trial
SMD: standardized mean difference
SPIRIT: Standard Protocol Items: Recommendations for Interventional Trials
TAU: treatment as usual
UMIN: University Hospital Medical Information Network
UMIN-CTR: University Hospital Medical Information Network clinical trial registry
UWES: Utrecht Work Engagement Scale
WHO-CIDI: WHO-Composite International Diagnostic Interview
